# Comparative Study of the Tell-Show-Do Technique With and Without a Dental Simulation Game to Reduce Pain and Anxiety in Pediatric Dental Patients: A Randomized Controlled Trial

**DOI:** 10.7759/cureus.115989

**Published:** 2026-09-09

**Authors:** Pratiksha Patil, Sakshi Malik, Deepika Jauhari, Rubaab Brar, Prachi Deshmukh, Gauri Patil

**Affiliations:** 1 Pediatric Dentistry, Daswani Dental College and Research Center, Kota, IND; 2 Pediatric and Preventive Dentistry, Daswani Dental College and Research Center, Kota, IND; 3 Pedodontics and Preventive Dentistry, Daswani Dental College and Research Center, Kota, IND; 4 Pediatric Dentistry, Dr. Hedgewar Smruti Runga Seva Mandal (HSRSM) Dental College and Hospital, Hingoli, IND

**Keywords:** behaviour management, dental anxiety, dental simulation game, pain management, pediatric dentistry, randomized controlled trial, tell-show-do

## Abstract

Background: Dental anxiety and pain in pediatric patients remain significant barriers to effective treatment delivery. The Tell-Show-Do (TSD) technique is a widely accepted non-pharmacological behavior management strategy; however, its efficacy may be augmented by interactive digital interventions such as dental simulation games. This study aimed to compare the effectiveness of the TSD technique with and without a dental simulation game in reducing pain and anxiety among pediatric dental patients.

Methods: This parallel-group randomized controlled trial enrolled 68 children aged 4-10 years requiring dental treatment (pulp therapy or extraction). Participants were randomly allocated into two groups: the control group (n = 34) received the TSD technique alone, while the intervention group (n = 34) received the TSD technique supplemented with a dental simulation game (BabyBus). Pain was assessed using the Wong-Baker Faces Pain Rating Scale (WBFPRS) and the Modified Toddler-Preschooler Postoperative Pain Scale (MTPPPS). Anxiety was evaluated through pulse rate - at baseline, during, and after treatment/procedure. Data were analyzed using the chi-squared test and independent-samples t-test, with statistical significance set at p ≤ 0.05.

Results: The intervention group had a lower mean WBFPRS score than the control group (3.12 ± 1.92 versus 4.47 ± 2.31), with a mean difference of 1.35 points (95% CI: 0.32-2.38; p = 0.011). MTPPPS scores did not differ significantly between the intervention and control groups (3.98 ± 1.73 versus 3.86 ± 1.40; mean difference: −0.12, 95% CI: −0.88 to 0.64; p = 0.757). Baseline pulse rates were comparable between the groups (81.00 ± 5.85 versus 79.67 ± 6.20 beats/min; p = 0.539). During treatment, the intervention group had a lower mean pulse rate than the control group (84.33 ± 7.10 versus 97.00 ± 8.40 beats/min; p < 0.001). No significant between-group difference was observed after treatment (73.67 ± 5.40 versus 74.67 ± 5.90 beats/min; p = 0.501).

Conclusion: The addition of a dental simulation game to the conventional TSD technique significantly reduced pain and anxiety in pediatric dental patients aged 6-10 years. Simulation-based pre-exposure may serve as a valuable adjunct to standard behavior management, particularly for technologically adept children of Generation Alpha.

## Introduction

Pain and anxiety during dental procedures constitute one of the most prevalent and challenging concerns in pediatric dentistry, often leading to uncooperative behavior, treatment avoidance, and long-term dental phobia [1-5]. Children frequently perceive the dental environment as unfamiliar and threatening, and their limited coping mechanisms predispose them to heightened emotional and physiological stress responses [6-8]. Such negative experiences during formative years can establish enduring patterns of dental avoidance that persist into adulthood, underscoring the clinical importance of effective pediatric behavior management [9-11].

To mitigate procedural anxiety and facilitate cooperation, a range of non-pharmacological behavior management techniques have been developed and refined over the decades [12]. Among these, the Tell-Show-Do (TSD) technique remains the most universally accepted and widely practiced approach, serving as a cornerstone of pediatric behavior guidance [13]. The TSD technique involves verbally explaining the upcoming procedure in age-appropriate language (Tell), demonstrating the procedure using instruments or models (Show), and then performing the procedure as described (Do). Its simplicity, non-invasiveness, and high parental acceptance have established TSD as the gold standard in pediatric dental behavior management [6,14-18].

Despite its widespread adoption, the TSD technique is not without limitations. Some studies have reported that the demonstration phase of TSD may paradoxically heighten anxiety in certain children, particularly when they are confronted with unfamiliar dental instruments or sounds for the first time [17]. Furthermore, the evolving landscape of pediatric behavior management necessitates the integration of contemporary strategies that align with children’s changing developmental needs and technological familiarity [15,19]. The acceptability and effectiveness of various behavior guidance techniques continue to be evaluated across diverse cultural and clinical settings [18,20].

In recent years, digital technology-based interventions have emerged as promising adjuncts to conventional behavior management strategies in pediatric dentistry [2,14]. Audio-visual distraction techniques, including animated cartoons, virtual reality, and mobile application-based games, have demonstrated variable but encouraging results in reducing procedural anxiety and pain perception [11,14,21]. Dental simulation games, in particular, offer an interactive and engaging platform that allows children to familiarize themselves with the dental environment, instruments, and procedures in a playful, non-threatening context [5,6]. By simulating the dental experience - enabling children to assume the role of a dentist and virtually explore instruments such as scalers, handpieces, and suction devices - these games may effectively reduce the element of surprise and provide cognitive pre-exposure to procedural stimuli [5,10,21-24].

The contemporary cohort of pediatric patients, frequently termed Generation Alpha (born from approximately 2010 onward), exhibits an unprecedented familiarity with digital devices and interactive media from a very early age [22]. For these technologically native children, a simulation game that mirrors the dental experience may offer a uniquely effective preparatory tool, enhancing their sense of control and reducing anticipatory fear prior to actual dental treatment [5,6,10]. Despite this conceptual promise, the evidence base comparing the combined effect of TSD with dental simulation games against TSD alone remains limited, and further well-designed clinical trials are warranted [15].

Therefore, the present study was designed as a parallel-group randomized controlled trial to evaluate the comparative effectiveness of the TSD technique with and without a dental simulation game (BabyBus: “BabyBus For Kids, For the Future”; www.babybus.com) in reducing pain and anxiety among pediatric dental patients aged 4-10 years undergoing pulp therapy or extraction.

## Materials and methods

Study design and ethical approval

This study was designed as a parallel-group, randomized controlled trial conducted in a pediatric dental clinical setting. Ethical approval was obtained from the Institutional Ethics Committee of the Department of Pediatric and Preventive Dentistry, Daswani Dental College and Research Center, Kota, prior to commencement of the study. The study protocol was explained in detail to the parents or legal guardians of all potential participants, and written informed consent was secured before enrolment. The trial was conducted in compliance with the Declaration of Helsinki and applicable ethical guidelines for research involving minors.

Study population and sample size

A total of 68 pediatric patients aged 4-10 years, reporting for dental treatment, were enrolled in the study. The participants were randomly allocated into two groups of 34 each using a simple randomization procedure. Group 1 (control group) received behavior management through the conventional TSD technique alone, while Group 2 (intervention group) received the TSD technique supplemented with a dental simulation game prior to the procedure (Figure 1).

**Figure 1:**
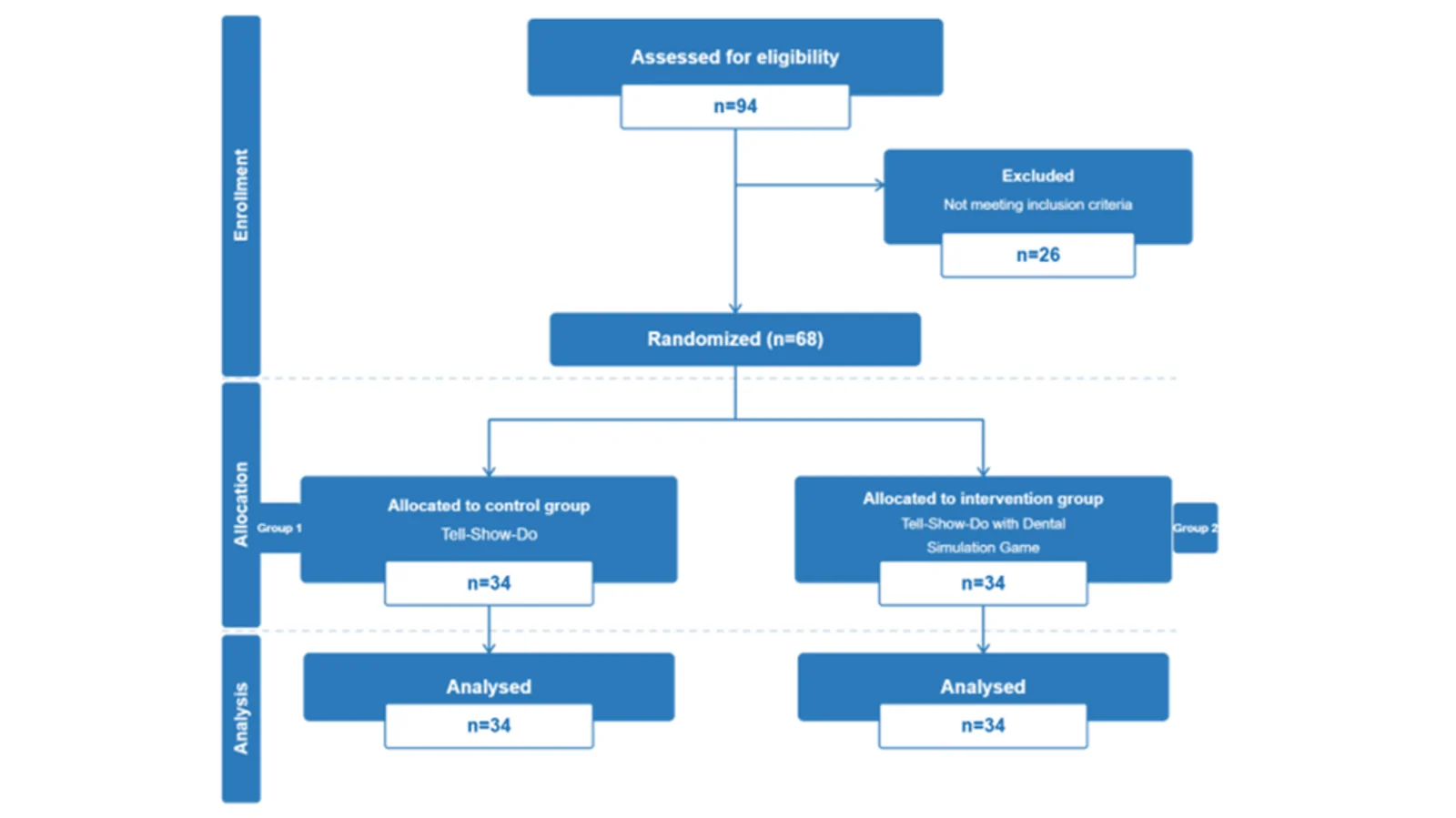
Participation selection protocol

The sample-size assessment was based on the primary outcome for this study. The observed mean difference between the control and intervention groups was 1.35 points, with a pooled standard deviation (SD) of 2.124, corresponding to a standardized effect size of 0.636. At a two-sided α of 0.05, 80% power, and equal group allocation, an exact independent-samples t-test calculation indicated that 40 participants per group (80 overall) would be required. After allowing for 10% attrition, the estimated recruitment target was 45 participants per group (90 overall). The final sample of 68 participants provided approximately 73% power for the observed effect.

Inclusion and exclusion criteria

The inclusion and exclusion criteria for this study are mentioned in Table 1.

**Table 1:** Inclusion and exclusion criteria for this study

Category	Criteria
Inclusion criteria	Children aged 4-10 years scheduled for dental treatment (pulp therapy or extraction)
	Children rated as "definitely positive" or "positive" on Frankl's Behavior Rating Scale
	Children who understood English and/or Hindi adequately to comprehend instructions and assessment scales
Exclusion criteria	Children with known systemic, mental, or oral pathological conditions that could influence pain perception or behavior
	Children rated as "definitely negative" or "negative" on Frankl's Behavior Rating Scale
	Children with a history of previous dental visits
	Children unfamiliar with smartphone-based games

Randomization and allocation

Eligible participants were randomly assigned to either the control group or the intervention group using a simple randomization method. Allocation concealment was maintained using sequentially numbered, sealed opaque envelopes to minimize selection bias.

Intervention protocol

In the control group (Group 1), each child received the standard TSD technique as the sole behavior management strategy. The clinician verbally explained the upcoming dental procedure using simple, age-appropriate language, demonstrated the procedure using dental instruments in a non-threatening manner, and subsequently performed the treatment.

In the intervention group (Group 2), children received the TSD technique as described above, supplemented with a dental simulation game (BabyBus) prior to the commencement of treatment. The game allowed children to interactively simulate a dental visit, including selecting an animated animal character requiring dental treatment, performing procedures such as dental scaling with an ultrasonic scaler, rinsing with a water gun, suctioning, dental radiography, cavity preparation with a dental drill, anesthesia administration, tooth filling, polishing, and crown placement. The game concluded with a positive feedback animation depicting the character’s happy response following treatment, thereby reinforcing a favorable perception of dental care. Sessions lasted a mean of 5.9 ± 0.8 minutes (range 4.7-7.8) and ran once only, in the waiting area, immediately before the child was seated. The module simulates examination, scaling, radiography, cavity preparation, local anesthesia, restoration, polishing, and crown placement; it does not depict extraction, so children scheduled for extraction were familiarized with the setting, the instruments, and the experience of local anesthesia rather than with the procedure itself.

Outcome measures

Pain and anxiety were assessed using the following validated instruments: (1) Wong-Baker Faces Pain Rating Scale (WBFPRS): a self-report subjective pain assessment tool consisting of six faces ranging from a smiling face (score 0: no hurt) to a crying face (score 10: hurts worst), widely validated for use in pediatric populations [4,5]; (2) Modified Toddler-Preschooler Postoperative Pain Scale (MTPPPS): a non-verbal, observer-rated behavioral pain scale assessing domains including verbal/vocal response, facial expression, body posture, and specific behaviors indicative of pain or distress [5]; and (3) pulse rate: physiological pulse rate (beats per minute) was recorded as an objective measure of anxiety at three time points: baseline (in the waiting room prior to any intervention), during the procedure, and after the procedure.

The primary outcome was self-reported pain on the WBFPRS, recorded by the child within five minutes of completion, with the faces presented by an investigator who had not performed the treatment and who gave no verbal cue as to the expected answer. Observer-rated pain was scored on the MTPPPS by a single trained pediatric dentist who observed the whole procedure. Pulse rate was taken as a 60 s fingertip pulse oximeter reading at three fixed points: in the waiting room before any intervention, during the operative phase, and immediately after completion. Before recruitment began, the MTPPPS observer was calibrated against a second examiner on 15 children not entered into the trial; agreement was good (intraclass correlation coefficient 0.88, 95% CI: 0.68-0.96).

Data collection procedure

At baseline, each child’s pulse rate was recorded in the waiting room using a digital pulse oximeter. For children allocated to the intervention group, the dental simulation game was administered on a smartphone device prior to treatment. Subsequently, dental treatment (pulp therapy or extraction) was carried out in both groups. During the procedure and after the procedure, pulse rate was recorded again. Upon completion of the procedure, pain was assessed using the WBFPRS and MTPPPS.

Hypothesis

The null hypothesis was that the groups would not differ in post-treatment WBFPRS score, post-treatment MTPPPS score, or pulse rate at any measured time point. The alternative hypothesis was that at least one of these outcomes would differ between groups.

Statistical analysis

Descriptive statistics were computed and expressed as mean ± SD. The chi-squared test was used for comparison of categorical variables between groups. The independent-samples t-test was employed for comparison of continuous variables (pain scores, pulse rate) between the control and intervention groups. A p-value of ≤0.05 was considered statistically significant. All statistical analyses were performed using appropriate statistical software. Analyses were performed in IBM SPSS Statistics version 26.0 (IBM Corp., Armonk, NY, USA).

## Results

There were no statistically significant between-group differences in baseline demographics, behaviors, procedures, and medical conditions (all p-values > 0.05), as shown in Table 2.

**Table 2:** Baseline demographic and clinical characteristics TSD: Tell-Show-Do; SD: standard deviation; dmft: decayed, missing, and filled teeth

Characteristic	Control (TSD alone)	Intervention (TSD + game)	p
Age (years), mean ± SD	7.23 ± 1.47	7.07 ± 1.40	0.660
Observed range	4.2-10.0	4.5-9.6	-
4-6 years, n (%)	14 (41.2)	17 (50.0)	-
7-8 years, n (%)	16 (47.1)	15 (44.1)	-
9-10 years, n (%)	4 (11.8)	2 (5.9)	-
Sex, male, n (%)	23 (67.6)	18 (52.9)	0.215
Sex, female, n (%)	11 (32.4)	16 (47.1)	-
Weight (kg), mean ± SD	22.71 ± 4.85	22.59 ± 4.22	0.909
dmft score, mean ± SD	5.32 ± 1.64	4.77 ± 2.19	0.247
Frankl rating 4 (definitely positive), n (%)	14 (41.2)	12 (35.3)	0.618
Frankl rating 3 (positive), n (%)	20 (58.8)	22 (64.7)	-
Procedure: pulp therapy, n (%)	22 (64.7)	25 (73.5)	0.431
Extraction, n (%)	12 (35.3)	9 (26.5)	-
Arch treated: mandibular, n (%)	19 (55.9)	18 (52.9)	0.808
Maxillary, n (%)	15 (44.1)	16 (47.1)	-
Parent present in operatory, n (%)	27 (79.4)	32 (94.1)	0.074
Morning appointment, n (%)	23 (67.6)	18 (52.9)	0.215
Baseline pulse rate (bpm), mean ± SD	81.16 ± 7.50	78.67 ± 8.16	0.195
Local anesthetic volume (mL), mean ± SD	1.47 ± 0.16	1.46 ± 0.21	0.941
Treatment duration (min), mean ± SD	25.20 ± 5.36	24.39 ± 4.48	0.502

As shown in Table 3 and Figure 2, respectively, in the case of self-reporting of pain intensity, a significant decrease was observed with a combination of TSD and simulation games as compared to only TSD (3.12 ± 1.92 versus 4.47 ± 2.31; the mean difference was 1.35, 95% CI of 0.32-2.38; p = 0.011). No differences in observer's evaluation of MTPPPS scores were reported between the groups (p = 0.757).

**Table 3:** Comparison of pain scores between the control group (TSD alone) and the intervention group (TSD + dental simulation game) Mean scores reported. Statistically significant (p ≤ 0.05; independent-samples t-test) SD: standard deviation; TSD: Tell-Show-Do

Outcome	Control mean ± SD	Intervention mean ± SD	Mean difference (95% CI)	t (df = 66)	p
Wong-Baker Faces Pain Rating Scale	4.47 ± 2.31	3.12 ± 1.92	1.35 (0.32 to 2.38)	2.63	0.011
Modified Toddler-Preschooler Postoperative Pain Scale	3.86 ± 1.40	3.98 ± 1.73	−0.12 (−0.88 to 0.64)	−0.31	0.757

**Figure 2:**
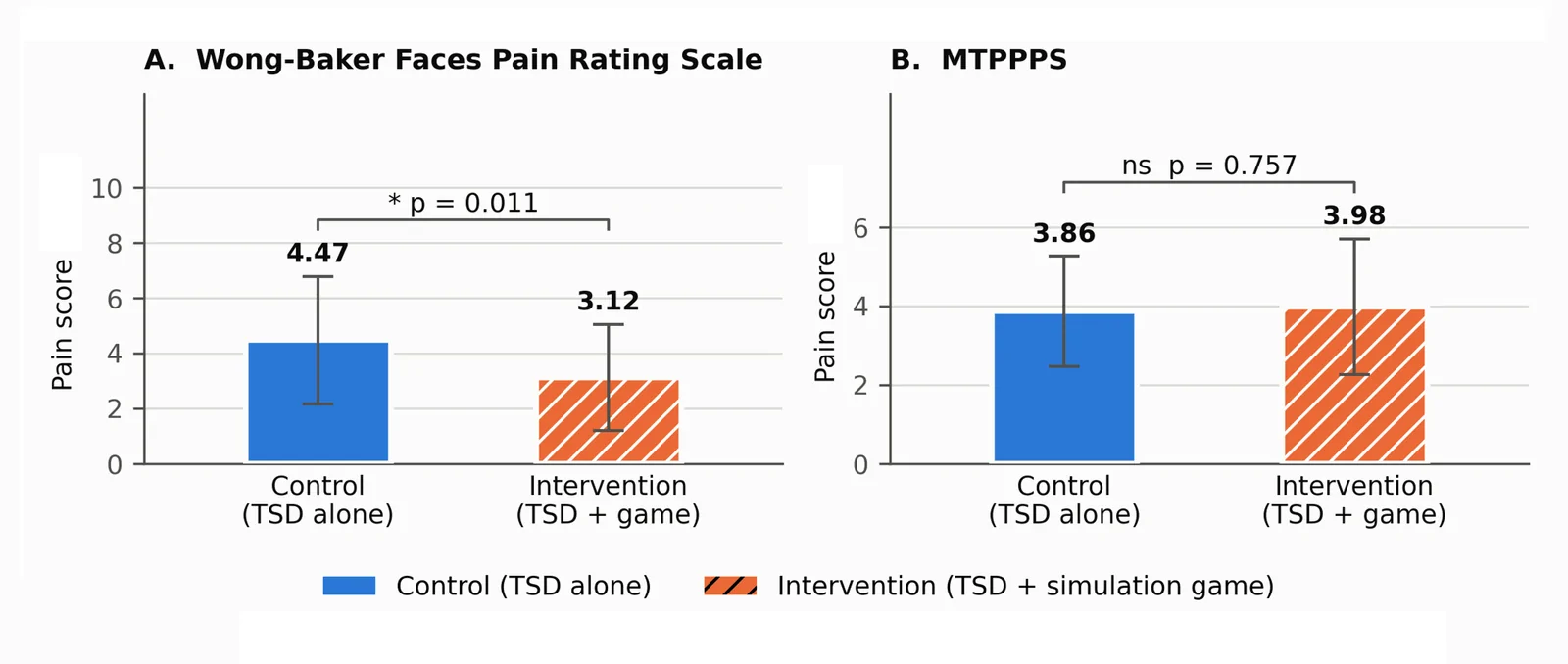
(A, B) Graphical representation of comparison of pain scores between the control group (TSD alone) and the intervention group (TSD + dental simulation game) TSD: Tell-Show-Do; MTPPPS: Modified Toddler-Preschooler Postoperative Pain Scale

The physiological measure of anxiety, assessed through pulse rate, demonstrated that baseline pulse rates recorded in the waiting room were comparable between the two groups, confirming the adequacy of the randomization process (Table 4 and Figure 3, respectively). The findings reveal that for the control group, there was a significant rise in their heart rate up to 97.00 bpm, while in the intervention group, there was a comparatively smaller mean heart rate value of 84.33 bpm; this indicated a significant difference at p-value < 0.05, which meant that the intervention had caused lowered physiological anxiety levels among participants in the study. The average heart rates after the treatment/procedure were significantly lower compared to before, with the average values being 74.67 and 73.67 bpm, respectively, for the two groups (p ≤ 0.05).

**Table 4:** Comparison of pulse rate (beats per minute) between the control and intervention groups at baseline and during treatment ^‡^Baseline values comparable between groups, indicating effective randomization *Mean values reported ^†^Statistically significant (p ≤ 0.05; independent-samples t-test)

Time point	Group 1 (control), mean ± SD*	Group 2 (intervention), mean ± SD*	t (df)	Cohen’s d	p-value
Baseline (waiting room)	79.67 ± 6.20	81.00 ± 5.85	0.62 (66)	0.16	0.539^‡^
During treatment	97.00 ± 8.40	84.33 ± 7.10	6.24 (66)	1.61	<0.001^†^
After treatment/procedure	74.67 ± 5.90	73.67 ± 5.40	0.68 (66)	0.18	0.501

**Figure 3:**
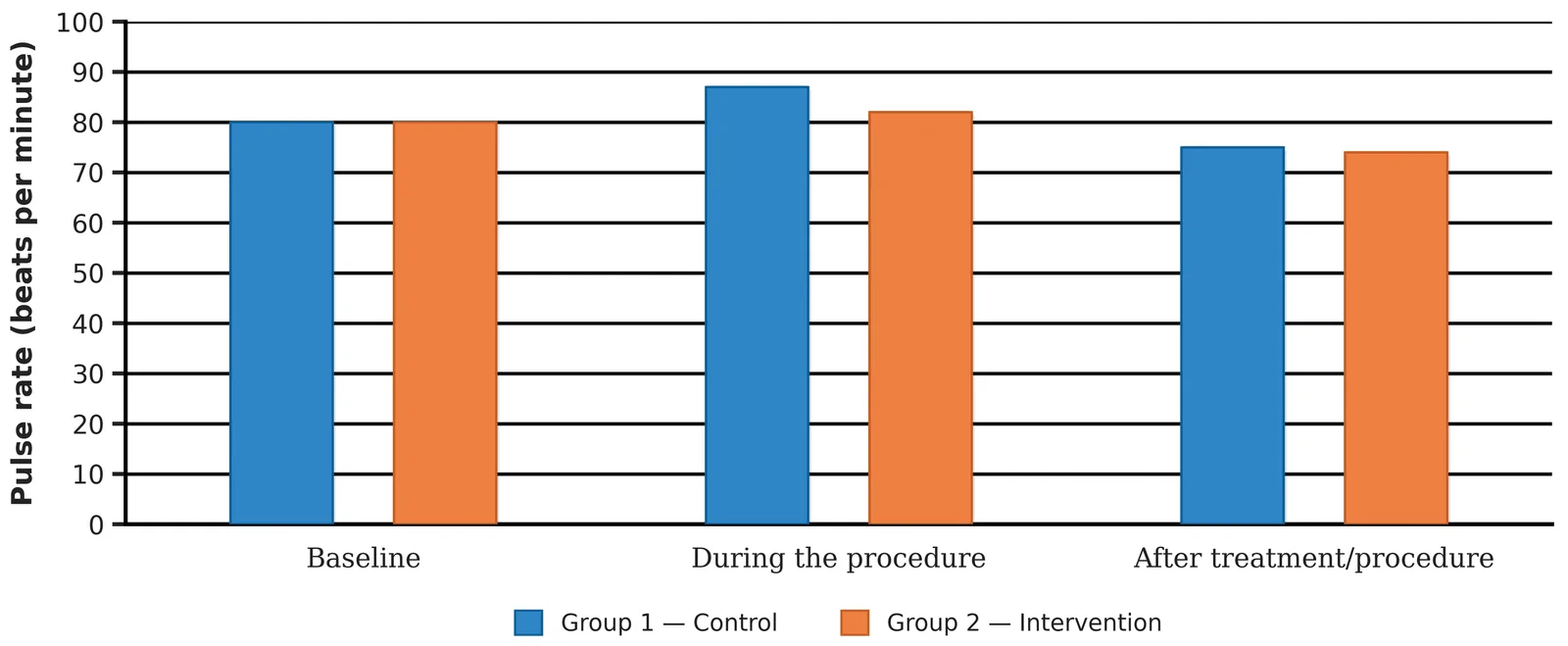
Graphical representation of comparison of pulse rate (beats per minute) between the control and intervention groups at baseline, during treatment, and after the procedure

## Discussion

The management of dental anxiety and pain in pediatric patients remains a central concern in contemporary pediatric dentistry, given the well-established association between negative childhood dental experiences and long-term dental avoidance behavior [13,15,24]. The present randomized controlled trial demonstrated that the combination of the TSD technique with a dental simulation game resulted in significantly lower pain and anxiety levels compared with TSD alone, supporting the integration of digital interactive tools into pediatric behavior management protocols.

The TSD technique has long been regarded as the gold standard for behavior management in pediatric dentistry due to its simplicity, non-invasiveness, and universal applicability [1,4,8,12]. Multiple studies have confirmed the efficacy of TSD in reducing dental anxiety and improving cooperation across diverse pediatric populations [3,9,19,25-30]. However, some investigations have suggested that TSD may have limited additional benefit in certain clinical scenarios or may inadvertently heighten anticipatory anxiety when children are visually confronted with dental instruments for the first time [7,17]. Patil et al. [9] reported that reframing techniques, when compared with TSD, can offer comparable or superior anxiety reduction, highlighting the potential for augmenting TSD with complementary strategies.

The findings of the present study are consistent with the growing body of evidence supporting the use of dental simulation games as effective adjuncts to conventional behavior management [5,6,10]. Karkoutly et al. [5] conducted a randomized clinical trial evaluating a dental simulation game against TSD for primary molar pulpotomy and reported a significant reduction in both pain and anxiety in the simulation game group, closely mirroring the outcomes of the present investigation. Similarly, Aziz and Jafar [6] demonstrated that the “Little Lovely Dentist” mobile application significantly alleviated dental anxiety in Iraqi children compared with TSD alone. Shah et al. [10] further corroborated these findings with the self-designed “Hop-A-Dent” game, which demonstrated significant anxiety reduction in children aged 6-10 years when compared with TSD.

The mechanism underlying the efficacy of simulation games likely involves cognitive pre-exposure and desensitization [5,26]. By allowing children to interactively engage with simulated dental procedures - including drilling, scaling, suctioning, and crown placement - in a playful, consequence-free digital environment, these games reduce the novelty and unpredictability of the actual clinical experience [6,10]. This pre-exposure may attenuate the fear-of-the-unknown component that is often a primary driver of pediatric dental anxiety [15,29]. Naik et al. [26] similarly reported the efficacy of the Tell-Play-Do approach in anxiety reduction, suggesting that incorporating play-based elements into preparatory strategies enhances their effectiveness.

The observation that pain perception was significantly lower in the intervention group is noteworthy, as it aligns with the well-documented bidirectional relationship between anxiety and pain in pediatric patients. Anxious children tend to perceive procedural pain more intensely due to heightened sympathetic activation and lowered pain thresholds [13]. Bhowmick et al. [2] reported that audio-visual distraction techniques significantly reduced dental anxiety compared with TSD alone, while Üstün et al. [14] demonstrated the efficacy of cartoon-assisted distraction in lowering both anxiety and pain during pediatric dental treatment. These findings collectively suggest that multisensory or interactive pre-treatment interventions may address both the cognitive and emotional dimensions of the pain-anxiety interplay more effectively than verbal behavior management alone.

The present study is particularly relevant in the context of the evolving demographic profile of pediatric patients. Children of Generation Alpha, born from approximately 2010 onward, exhibit a high degree of familiarity and comfort with digital technologies, making simulation game-based interventions especially appropriate for this cohort [29,30]. As Mistry et al. [29] noted, the shifting trends in pediatric behavior management necessitate the adoption of technology-driven strategies that resonate with the digital nativity of contemporary children. Several recent systematic reviews and meta-analyses have highlighted the growing evidence supporting non-pharmacological digital interventions in managing pediatric dental anxiety [25,28].

The broader context of parental acceptance also supports the integration of technology-based behavior management tools. Massignan et al. [23] reported high parental acceptance of non-invasive, distraction-based behavior management techniques, while Gómez et al. [18] further highlighted cross-cultural variability in parental acceptance, underscoring the need for culturally sensitive behavior management approaches. The observed findings reflect the broader understanding that quantifying pediatric pain and discomfort is crucial for optimizing clinical compliance [31].

Several limitations of the present study warrant acknowledgment. First, the absence of operator blinding, inherent to the nature of the intervention, may introduce performance bias. Second, the specific cultural and linguistic context of the study population may limit the generalizability of findings to other settings. Third, the exclusion of children with prior dental experience restricts the applicability of results to treatment-naïve populations. Fourth, the study assessed immediate post-procedural outcomes only; long-term effects on dental anxiety and subsequent visit compliance were not evaluated. Finally, the use of a single dental simulation game (BabyBus) precludes conclusions regarding the comparative efficacy of different simulation platforms.

Future research should incorporate multicenter designs with larger sample sizes, longer follow-up periods, and diverse simulation platforms to establish the robustness and generalizability of these findings. Investigations comparing simulation games with other emerging behavior management modalities, such as virtual reality, cognitive behavioral play therapy, and puppet-based interventions, would further enrich the evidence base.

## Conclusions

With all due consideration to the restrictions of the present single-center study, the introduction of a simulation game into the classic TSD approach brought a significant reduction in the child’s subjective assessment of pain, along with the lower pulse rate observed while performing the treatment procedures in the group of four to 10-year-olds. It should be noted, however, that no difference was detected when comparing observer-assessed pain and pulse rate scores at the post-procedural time point. In this regard, the use of simulation as an effective pre-exposure technique proved to be effective in terms of subjective evaluation of pain as well as physiological reaction. Further adequately powered, multicenter studies incorporating validated anxiety measures, procedure-specific analyses, and longer follow-up are needed before routine clinical implementation can be recommended.
